# Problems of Sanitation

**Published:** 1921-02-05

**Authors:** 


### PROBLEMS OF SANITATION.

Mr. Hoskins, the new President of the Institution of Sanitary Engineers, has presented a not very encouraging review of the progress of public health in this country. He admits that as a direct outcome of the war we have become better acquainted with the hygienic conditions of the nation, but he seems to think that our advance in some directions in the control of health matters has been counter-balanced by "retrograde changes of a very deplorable nature in others." It is difficult to discover in any of the published reports of Mr. Hoskin's statements to what retrograde movements he specially refers in this sweeping assertion. From the extensive legislation which has been passed to prevent the pollution of streams, to regulate the construction of new streets and buildings, and to ensure healthy conditions in dwellings and factories, it would appear, he remarks, that the sanitary condition of the country must be extremely good. Then he proceeds to ask, But are the laws and by-laws in this respect strictly enforced and properly applied? Have we brought our practice up-to-date with our theoretical knowledge? If so, "it is somewhat disappointing that the percentage of sickness in the country is so great."

This is surely a lame and not a very helpful conclusion. Of course, there is still a large percentage of sickness in this country, but it is considerably less in many important respects than it was, and there is enough evidence to satisfy a blind man that very definite results beneficial to the health of the people have been obtained solely through modern sanitary legislation. Again, it is not altogether to be expected that we should have brought our practice up to date with our theoretical knowledge. Practice is rarely abreast of theory in any department of human activity; and, after all, it is to the great body of sanitary engineers, and particularly to those whose work is concerned with the administration of local authorities, that the public is entitled to look for guidance in bringing practice more in line with theory. It is also upon sanitary engineers engaged in the service of the public that the responsibility chiefly rests to see that the laws and by-laws are "strictly enforced and properly applied." They must share in a large measure of the blame if the contrary is the case, as they must if it is true that we are in the midst of "retrograde changes of a very deplorable nature." We do not believe that, generally speaking, it is true, and Mr. Hoskins should be called upon to give chapter and verse for a statement which casts a serious reflection upon the public-health services of the country.

Sanitation is a progressive science, and we are a very long way from the ideal. But in recent years real progress has been made, whether in the disposal of sewage or refuse, in the purification of water supply, in the planning of streets or houses, in the manner in which infectious disease is dealt with, or in many other important directions. When Mr. Hoskins' strictures are reduced to their proper proportions they are probably all contained in his complaint that in some of the smaller districts sanitary Acts and by-laws "appear to have been entirely ignored"—we would prefer to say, badly administered. It is natural that the larger towns should have a much more efficient administration, and there is a good deal to be said for the argument that, with regard especially to sanitary arrangements, small adjacent districts should be grouped wherever possible—and it is possible in many of the populous areas of the Midlands and the North—with the large central authority. In so far as Mr. Hoskins intended his jeremiad to be a call for greater energy and enterprise in matters of sanitation, it may have value; but its usefulness is certainly not enhanced by an unconscious exaggeration of the prevailing conditions.

To every person who is interested in these matters, and whose memory takes them back even a few years, it must be obvious that considerable progress and enterprise have been shown by the great majority of local authorities, not only in the purely scientific improvement of sanitation in their districts but also in the financing and economical running of these schemes. If any person is led to draw from Mr. Hoskins' words the inference that England lags behind other nations in the science and practice of sanitation, let him spend a week in any town in any part of France.

